# Bullet Appendicitis: An Unusual Cause to a Rather Straight-Forward Diagnosis

**DOI:** 10.7759/cureus.16638

**Published:** 2021-07-26

**Authors:** Abdullah R Khazindar, Rawan A Thabit, Arwa Badeeb

**Affiliations:** 1 Department of Radiology, University of Jeddah - College of Medicine, Jeddah, SAU; 2 Radiology, King Abdulaziz University, Jeddah, SAU

**Keywords:** foreign body, gun bullet, acute appendicitis, ct (computed tomography) imaging, right iliac fossa

## Abstract

A 19-year-old male patient presented to the emergency department (ED) with pain in the right iliac fossa. Computed tomography (CT) scan of the abdomen and pelvis revealed signs of acute appendicitis, as a result of a metallic foreign body beyond the appendiceal orifice. Upon further questioning, the patient gave a history of ball bearing (BB) gun bullet ingestion in the past. Although rare, foreign body appendicitis occurs. A radiologist should be mindful to reporting such cases especially bizarre foreign bodies for example bullets as it may warrant psychiatric consultation or alter surgical management.

## Introduction

Acute appendicitis is one of the most common causes of abdominal pain - in both pediatric and adult patients - that requires visiting the emergency department [1]. Although most of the patients have typical presentations, others present atypically which makes it challenging for clinicians to reach the correct diagnosis. Patients classically present with generalized abdominal pain that shifts to the right iliac fossa (RIF). This pain is usually associated with nausea, vomiting, fever, and leukocytosis. Although acute appendicitis diagnosis starts clinically, it requires radiological studies to confirm the diagnosis, look for complications, and to exclude another differential [2]. 

Foreign body ingestion may cause complications in the alimentary tract. One of the rare complications is acute appendicitis. It is even rarer to be caused by ingesting a bullet. In our case, we discuss a patient diagnosed with acute appendicitis after accidentally ingesting a ball bearing (BB) bullet.

## Case presentation

A 19-year-old male patient came to the ED with typical signs and symptoms of acute appendicitis. He gradually experienced generalized abdominal pain that localized to the right iliac fossa (RIF) after several hours. This was associated with a few episodes of nausea and vomiting. On examination, he was febrile with local tenderness in the RIF.

**Figure 1:**
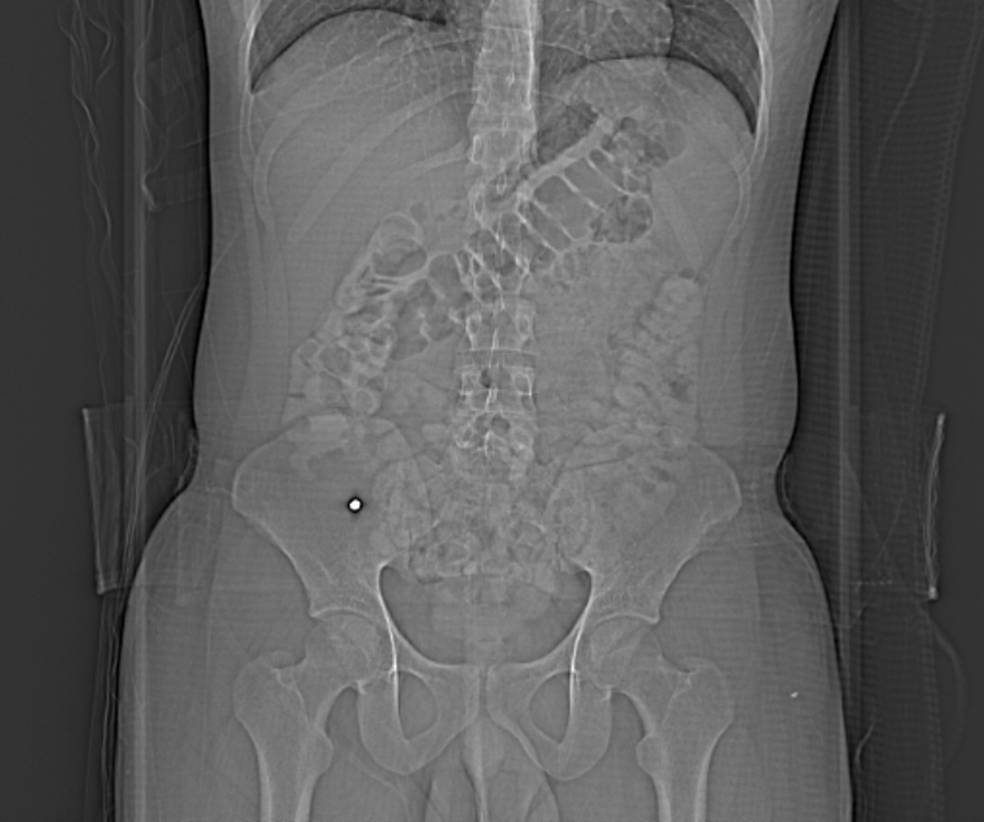
Scout Image The scout image of the CT scan with a round metallic density foreign body in the right lower quadrant

**Figure 2:**
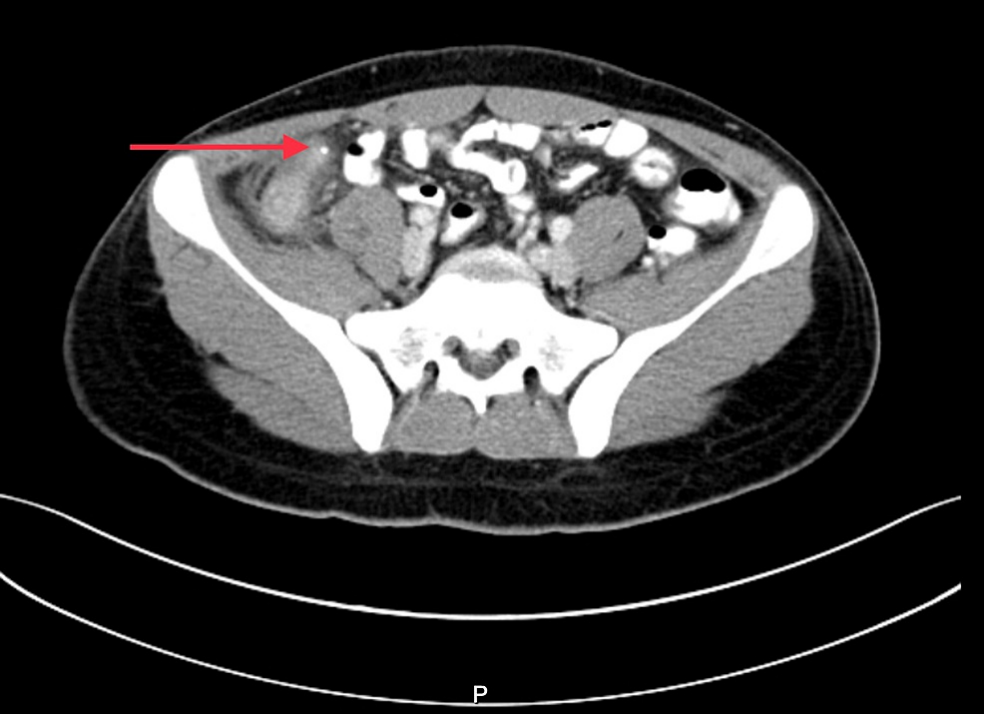
Axial CT Image of the Lower Abdomen With Intravenous and Oral Contrast Shows an inflamed thickened appendix with mucosal enhancement and peri-appendiceal fat stranding. Deep into the appendix is a metallic dot that is incompletely seen on the image

**Figure 3:**
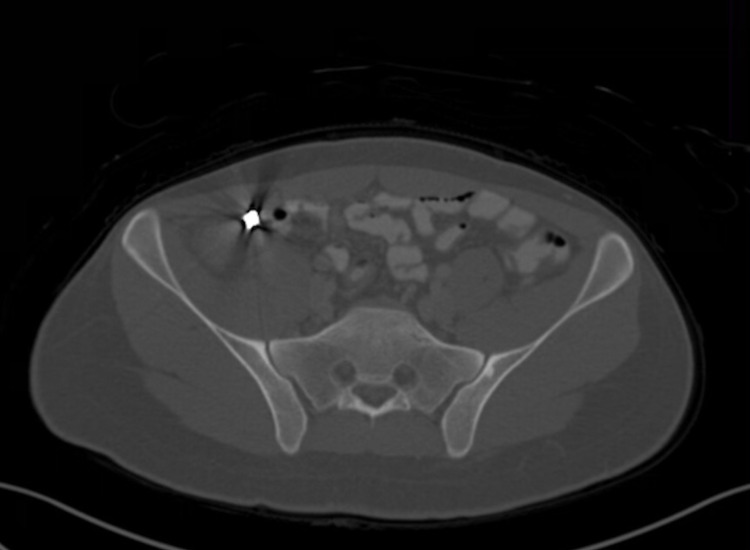
Bone Window of CT Scan Shows the entirety of the metallic foreign body causing streak artifact.

CT scan of the abdomen and pelvis was performed with administration of oral and intravenous (IV) contrast. The scout images showed a well-defined high-density structure in the right iliac fossa (Figure 1). The appendix was markedly dilated measuring 1.6 centimeters (cm) in thickness with peri-appendiceal fat stranding, and mucosal enhancement. There was reactive adenopathy in the right lower abdomen as well (Figure 2). A large well defined 0.6 cm metallic density was beyond the appendiceal orifice. Unlike typical appendicoliths, this was round man made, looking like a BB bullet (Figure 3). There were no signs of perforation or abscess formation. Upon further questioning, the patient reported accidentally ingesting an air gun BB bullet one month back. The patient underwent an emergent laparoscopic appendectomy without any intra-operative complications. The appendectomy specimen revealed the lodged within the appendix BB bullet. The patient had an uneventful postoperative course and was discharged home a few days later.

## Discussion

Appendicitis is of frequent ED presentation among the general population. Acute abdominal pain accounts for almost 7-10% of all ED visits. Of those, appendicitis constitutes 4.54% in patients aged < 65 years [1].

Appendicitis was a clinical diagnosis in the past. Patients mostly present with generalized abdominal pain that afterward shifts to the right iliac fossa. They frequently have nausea and vomiting. Radiographs, although of low diagnostic utility were sometimes ordered to check appendicoliths or assess for alternative diagnoses such as bowel obstruction. With the advancement of radiological technologies over the past decades, ordering CT of the abdomen and pelvis became essential to confirm the diagnosis, exclude complications or come up with an alternative diagnosis. Ultrasound remains a main diagnostic tool in pediatric patients and young women. 

The American College of Radiology (ACR) appropriateness criteria has provided many scenarios on the use of CT imaging to diagnose appendicitis. In the typical scenario when a patient is presenting with right lower quadrant pain, fever, and/or leukocytosis, contrast-enhanced CT in the adult age group is the initial and most appropriate imaging modality [2]. The CT imaging criteria for acute appendicitis include appendiceal thickening with greater than 6 mm, appendiceal wall thickening ≥ 3mm, hyperenhancement of the appendiceal wall, presence of an appendicolith, and intramural gas. Cecal changes such as cecal apical thickening, the cecal bar sign, and the arrowhead are best seen after rectal contrast administration. Peri-appendiceal inflammation includes peri-appendiceal fat stranding, thickening of the lateral conal fascia and mesoappendix, extraluminal fluid, phlegmon, abscess, and ileocecal lymph node enlargement can also be present. Some of the recognized complications of appendicitis include perforation, abscess formation, peritonitis, pylephlebitis, hepatic abscess, or gangrenous appendicitis [3].

Appendicitis is frequently caused by fecolith or lymphoid hyperplasia that causes obstruction of the appendiceal lumen. Cecal neoplasms are a concern in older patients. Foreign bodies, although rarely cause complications in the alimentary tract, may be a cause for appendicitis, as in our case [4].

A way to explain foreign body appendicitis is by the foreign body’s weight, layering dependently in the cecum, moving towards the appendiceal orifice -especially if retrocecal- which expands to allow the entry of the foreign body into its lumen [4]. Patients can remain asymptomatic or present with appendicitis after varying periods of time. In our case, the patient presented after a month of the bullet ingestion incident. 

Various foreign bodies were reported to cause appendicitis in the literature such as pins, needles, and screws [5,6,7]. These objects usually have sharp edges to elicit appendicitis with probable perforation unlike blunt objects as the BB bullet in our case. Bullet appendicitis is even rarer. To our knowledge, there were only a handful of appendicitis cases that were related to a gunshot or accidentally swallowing a bullet or its fragment.

Bullets have many sizes and varied shapes depending on their intended use [8]. These factors affect the chances of lodgment into the appendix. In our case, the BB bullet was small enough (6 mm) that it advanced through the appendiceal orifice.

Bullets are manufactured from different materials such as lead, steel, bismuth, etc. They may be coated by an outer material such as a lead bullet coated by a copper jacket or plastic [8]. A rare but feared bullet lodgment complication anywhere in the human body is lead poisoning. Looking at the literature, a 45-year-old male with a history of schizophrenia presented with laboratory findings of severe lead poisoning after ingesting 206 lead bullets [9]. This case probably happened over a long period of time and the number of bullets likely played role in the eventual toxicity. Thankfully, our patient didn’t have lead poisoning as he presented a month after single bullet ingestion.

Foreign body ingestions can be seen among different patient groups such as children or even adults who may suffer from substance abuse or psychiatric disorders. Those participating in dangerous sporting events such as hunting may rarely ingest a foreign body by accident such as in our patient [10].

## Conclusions

A radiologist should be mindful of the presence of uncommon causes of common diagnoses such as appendicitis. Reporting the presence of bizarre foreign bodies is important, as it may lead clinicians to investigate if a psychiatric health issue is an underlying concern or alter their surgical plan to removing an inflamed foreign body containing an appendix.

## References

[1] Cervellin G, Mora R, Ticinesi A, Meschi T, Comelli I, Catena F, Lippi G (2016). Epidemiology and outcomes of acute abdominal pain in a large urban emergency department: retrospective analysis of 5,340 cases. Ann Transl Med.

[2] Garcia EM, Camacho MA, Karolyi DR (2018). ACR appropriateness criteria® right lower quadrant pain-suspected appendicitis. J Am Coll Radiol.

[3] Leite NP, Pereira JM, Cunha R, Pinto P, Sirlin C (2005). CT evaluation of appendicitis and its complications: imaging techniques and key diagnostic findings. AJR Am J Roentgenol.

[4] Antonacci N, Labombarda M, Ricci C, Buscemi S, Casadei R, Minni F (2013). A bizarre foreign body in the appendix: a case report. World J Gastrointest Surg.

[5] Hazer B, Dandin O, Karakaş DO (2013). A rare cause of acute appendicitis: an ingested foreign body. Ulus Travma Acil Cerrahi Derg.

[6] Benizri EI, Cohen C, Bereder JM, Rahili A, Benchimol D (2012). Swallowing a safety pin: report of a case. World J Gastrointest Surg.

[7] Sarkar RR, Bisht J, Sinha Roy SK (2011). Ingested metallic foreign body lodged in the appendix. J Indian Assoc Pediatr Surg.

[8] (2021). Forensic pathology of firearm wounds. https://emedicine.medscape.com/article/1975428-overview.

[9] McNutt TK, Chambers-Emerson J, Dethlefsen M, Shah R (2001). Bite the bullet: lead poisoning after ingestion of 206 lead bullets. Vet Hum Toxicol.

[10] Hunter TB, Taljanovic MS (2003). Foreign bodies. Radiographics.

